# Who is reporting non‐native species and how? A cross‐expert assessment of practices and drivers of non‐native biodiversity reporting in species regional listing

**DOI:** 10.1002/ece3.10148

**Published:** 2023-05-28

**Authors:** Andry Castro, Joana Ribeiro, Luís Reino, César Capinha

**Affiliations:** ^1^ Centro de Estudos Geográficos, Instituto de Geografia e Ordenamento do Território Universidade de Lisboa, Rua Branca Edmée Marques Lisboa Portugal; ^2^ CIBIO, Centro de Investigação em Biodiversidade e Recursos Genéticos, InBIO Laboratório Associado, Campus de Vairão Universidade do Porto Vairão Portugal; ^3^ BIOPOLIS Program in Genomics, Biodiversity and Land Planning CIBIO Vairão Portugal; ^4^ CIBIO, Centro de Investigação em Biodiversidade e Recursos Genéticos, InBIO Laboratório Associado, Instituto Superior de Agronomia Universidade de Lisboa Lisboa Portugal; ^5^ Laboratório Associado Terra Lisboa Portugal

**Keywords:** biodiversity data reporting, biogeographical changes, biological invasions, global changes, invasive alien species, non‐native taxa, species checklists

## Abstract

Each year, hundreds of scientific works with species' geographical data are published. However, these data can be challenging to identify, collect, and integrate into analytical workflows due to differences in reporting structures, storage formats, and the omission or inconsistency of relevant information and terminology. These difficulties tend to be aggravated for non‐native species, given varying attitudes toward non‐native species reporting and the existence of an additional layer of invasion‐related terminology. Thus, our objective is to identify the current practices and drivers of the geographical reporting of non‐native species in the scientific literature. We conducted an online survey targeting authors of species regional checklists—a widely published source of biogeographical data—where we asked about reporting habits and perceptions regarding non‐native taxa. The responses and the relationships between response variables and predictors were analyzed using descriptive statistics and ordinal logistic regression models. With a response rate of 22.4% (*n* = 113), we found that nearly half of respondents (45.5%) do not always report non‐native taxa, and of those who report, many (44.7%) do not always differentiate them from native taxa. Close to half of respondents (46.4%) also view the terminology of biological invasions as an obstacle to the reporting of non‐native taxa. The ways in which checklist information is provided are varied, but mainly correspond to descriptive text and embedded tables with non‐native species (when given) mentioned alongside native species. Only 13.4% of respondents mention to always provide the data in automation‐friendly formats or its publication in biodiversity data repositories. Data on the distribution of non‐native species are essential for monitoring global biodiversity change and preventing biological invasions. Despite its importance our results show an urgent need to improve the frequency, accessibility, and consistency of publication of these data.

## Graphical abstract

Each year, hundreds of species checklists are published. However, many of these checklists are difficult to identify, collect and integrate into analytical workflows, due to distinct data reporting structures, storage formats, the omission of relevant information or the use of ambiguous or inconsistent terminology. We conducted an online survey targeting authors of species regional checklists to identify the current practices and drivers of the geographical reporting of non‐native species in the scientific literature.

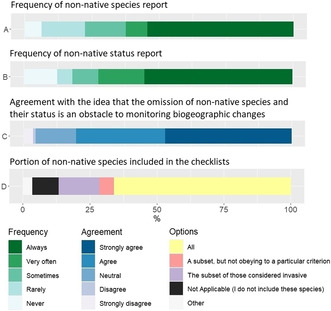

## 1 INTRODUCTION

Our planet is undergoing major biodiversity and biogeographical changes (Sala et al., 2000), for which the human‐mediated transportation and introduction of species outside of their native ranges is a major contributing factor (Pyšek et al., 2020; Seebens et al., 2017). While many of the introduced species fail to establish, some eventually succeed and become a permanent addition to local ecosystems. Altogether, these “established non‐native species” (hereafter referred to as “non‐native species,” for simplicity) already represent a substantial portion of biodiversity in many regions of the planet (McGeoch et al., 2010; Seebens et al., 2017), reshaping biogeographical patterns (Capinha et al., 2020), trophic networks (Bezerra et al., 2016), and the functioning of ecosystems (e.g., Wardle & Peltzer, 2017). In addition, a subset of non‐native species also causes important negative impacts to human well‐being, economy, and biodiversity (the so‐called “invasive alien species”; e.g., Gallardo et al., 2016; Shackleton et al., 2019; Zenni et al., 2021).

Despite the increasing presence of non‐native species in ecosystems worldwide (Seebens et al., 2017), obtaining a comprehensive, up‐to‐date description of their distribution remains, in general, a difficult task (Seebens et al., 2020). Knowledge about the distribution of these taxa is crucial to assess ongoing biodiversity changes and preventing negative impacts posed by invasive species (Brondízio et al., 2019); however, primary distribution data (i.e., records of the species occurrence) are usually scattered across distinct data sources of multiple types (e.g., scientific literature, biodiversity observation databases, technical documents, and gray literature), with each source often adopting distinct data reporting structures, publication formats and terminology. These issues resulted in a number of recent initiatives (e.g., Darwin Core—DwC, FAIR workflow) of aggregation and standardization of distribution data for non‐native species (Reyserhove et al., 2020; Wieczorek et al., 2012) and the development of technical pipelines aiming to automate the data assimilation and harmonization procedures (e.g., Seebens & Kaplan, 2022). Despite these efforts, a better understanding of the factors behind the lack of standardization of non‐native species reporting data remains necessary to act upstream of the problem and reduce reliance on post‐publication data aggregation efforts, which can be resource‐consuming and difficult to maintain in the long term.

One example of data sources where the issues of non‐native species reporting are most evident are species regional checklists. These checklists (sometimes also named simply as species “lists” or “inventories”) are a widely used approach to inform about the species that occur in a region at a particular time, ranging from simple, tabular‐type, listing of the species, to comprehensive, textual and image‐supported description of their local habitats, origin, and other attributes. Each year, hundreds of these checklists are published in peer‐reviewed journals, constituting one of the most widespread, and valuable sources of biodiversity information, enabling, for example, the direct quantification of key metrics of biodiversity change, such as species richness and taxonomic composition (Chase et al., 2019). However, several challenges hinder the widespread use of these data (Reyserhove et al., 2020), particularly concerning non‐native biodiversity. Difficulties include the uneven reporting of non‐native species among authors, with some reporting these but not always identifying them as non‐native, and others listing only native taxa. Furthermore, when non‐native species are listed and identified, authors often use inconsistent terminology, for example, the terms “introduced,” “alien,” “exotic,” “nonindigenous,” or “invasive” can appear as synonyms or to distinguish between establishment or nativity statuses (Pyšek et al., 2004). These issues add to others, not specific to non‐native species, including the widespread adoption of publication formats that are non‐machine‐reading friendly (e.g., Portable Document Format “PDF”), and the use of unstructured data reporting structures, which can include tables, descriptive text and a mix of both.

In this context, we here analyze the patterns of non‐native species reporting in regional species checklists and investigate factors potentially associated to the patterns observed. Specifically, we surveyed experts who authored or co‐authored species checklists in past years concerning: (1) the practices they adopted in reporting non‐native taxa, (2) their expertise and training background; and (3) perceived drivers for the practices they adopted. We analyze these data aiming to identify expert‐related factors that drive commonalities in species reporting practices and to highlight most relevant difficulties in further easing the access and harmonizing the biogeographical data that is being published for non‐native taxa.

## 2 MATERIALS AND METHODS

### 2.1 Survey approach

To investigate the practices and drivers of non‐native biodiversity reporting in species checklists, we conducted an online survey aimed at authors of these lists. To identify these authors, in November 2021, we performed a literature search on Google Scholar, employing the search words: (“Species” AND “Checklist”). Because our aim was to obtain a cross‐taxonomic global‐scale assessment, we did not employ any terms referring to geographical areas or species groups. We restricted the search to documents published between 2000 and 2021, from which we examined the title and abstract of each, to identify those effectively referring to species regional checklists (i.e., a publication aimed at listing all species of a taxonomic group or groups, known to be occurring in a geographical area). From the retained publications, we identified a total of 505 authors for which an e‐mail contact was provided (corresponding author or the first author when the identification of corresponding author was not possible; See Appendix S1). We sent an email inviting experts to participate in the survey, mentioning a response acceptance period from 8th March to 19th April 2022, totaling 6 weeks. During this period, we sent two reminders (March 29, 2022, at 03:07 a.m and April 12, 2022 at 09:00 a.m). In the first message, participants were also informed about the reason they were invited, the general aim of the survey and the guarantee of anonymity of responses. In this regard, the survey was checked and approved by the Ethics Committee of the Institute of Geography and Spatial Planning of the University of Lisbon.

### 2.2 Survey measures

The online survey was implemented in google forms, in English language and was divided into six sections, “A” to “E” plus an introductory section. The introductory section contained information about the aim of the study, estimated survey duration, ethics, and personal data protection information, and one mandatory question to confirm agreement to participate in the survey. In section A, we provided the definitions for the terms used in the survey, such as regional species checklist, established non‐native species and non‐native invasive species, and asked participants to confirm if they have published a species checklist in the past 20 years. Only those providing this confirmation were able to proceed to the following sections. In section B, we asked for a self‐assessment of expertise in distinct taxonomic groups (plants, vertebrates, invertebrates, microorganisms, and fungi), environmental realms (terrestrial, freshwater, and marine), and biomes of focus (tropical, subtropical, temperate, and polar). In section C, we asked about the species checklists published by the respondent, including number of those published and the geographical region(s) they referred to (based on the United Nations Subregions geoscheme from United Nations Statistics Division), taxonomic groups reported, and habitat represented. The last two sections (D and E) asked about the type and mode of non‐native species data reporting in the checklists. This included questions about the addition of non‐native established species in the checklists, of their non‐native status, issues related to terminology and definitions, how the information is supplied, including the provision of machine‐readable formats or publication in biodiversity data repositories (see Appendix S2). Most questions in the survey are close‐ended and use a Likert scale. This scale is commonly used to measure values, perceptions, attitudes, knowledge, and behavior (Likert, 1932; Ribeiro et al., 2019; Tiago et al., 2017). Where appropriate, we implemented a five‐point Likert scale of agreement (1 = strongly disagree, 2 = disagree, 3 = neutral, 4 = agree, 5 = strongly agree), frequency (1 = never, 2 = rarely, 3 = sometimes, 4 = very often, 5 = always), perceived knowledge (1 = no knowledge, 2 = little knowledge, 3 = moderate knowledge, 4 = good knowledge, 5 = expert‐level knowledge), research focus (1 = no focus, 2 = little focus, 3 = moderate focus, 4 = high focus, 5 = very high focus), expertise (1 = no expertise, 2 = little expertise, 3 = moderate expertise, 4 = high expertise, 5 = very high expertise), and quantities (1 = none, 2 = very few, 3 = some, 4 = most, 5 = all).

### 2.3 Statistical analysis

We used ordinal logistic regression models to analyze the relationships between respondent's expertise and research focus with the responses concerning: (1) frequency of non‐native reporting; (2) frequency of non‐native status differentiation; (3) knowledge about the terms and definitions referring to biological invasions and (4) the provision of data in machine‐readable formats or its publication in biodiversity data repositories. Ordinal regression was chosen because our independent variables are ordered variables representing Likert scales. In all models, we used the same set of predictors: respondent's self‐assessed level of expertise in each taxonomic group (plants, vertebrates, invertebrates, microorganisms, and fungi) and environmental realm (terrestrial, freshwater, and marine), and quantity of checklists published in each biome (tropical, subtropical, temperate and polar). We selected these predictors because we expect to find an effect for taxonomy, realms, and biomes in the patterns of response owing to uneven levels of knowledge across these (Collen et al., 2008; Troudet et al., 2017), leading to differences in the focus given to non‐native taxa. In other words, we expect that taxonomic groups and environments that have been historically better studied (e.g., vertebrates and terrestrial environments in temperate regions) will show a higher reporting of non‐native taxa and vice‐versa. Concerning non‐native status differentiation, the analysis used only the subset of respondents who mentioned to report non‐native species (i.e., responses “rarely” to “always”), totaling 105 answers. The ordinal logistic regressions were implemented in R programming language (R Core Team, 2022) through *Polr* function from *“mass”* package (Venables & Springer, 2002).

## 3 RESULTS

### 3.1 Background of respondents

Out of the 505 invitations sent via email, 42 were automatically returned due to reasons such as non‐existent email addresses, resulting a non‐contact rate of 8.3%. In total, we received 113 completed submissions (response rate = 22.4%), of which 112 were fully completed (99.1% of all submissions).

Concerning the taxonomic expertise of participants, most had no expertise in microorganisms and fungi (81.3% and 79.5%, respectively). On the other hand, invertebrates were the taxonomic group for which high or very high expertise was most prevalent (38.4% of all participants). Vertebrates and vascular plants showed intermediate levels of high to very high expertise (25% and 20.6%, respectively). Concerning environmental realms, the marine one had the highest proportion of respondents with “no expertise” (49.1%). On the other hand, the terrestrial realm had the highest prevalence of high to very high expertise (59.8%), followed by freshwater (23.2%), and marine areas (18.8%).

About 35.7% of respondents published between 2 and 3 checklists in the past 20 years, ~32% between 4 and 10 and ~20% more than 10. Most of respondents had a high focus or very high focus (31.3% and 37.5%, respectively) in reporting species at the country level or at sub‐national administrative divisions, followed by protected areas (e.g., National Parks, Reservations, etc.; 28.6% and 25%, respectively), and non‐protected, non‐urban habitats (e.g., wetlands, forests, coastal habitats; 28.6% and 17.9%, respectively). In contrast, little (26.8%) or no focus (33%) was given to human‐dominated habitats (e.g., urban areas, farmlands, orchards, etc.).

Considering the taxonomic groups reported in the checklists, respondents had a greater exclusive focus on invertebrates (33.9%) and lowest in microorganisms and fungi (92.9% indicate no checklist published on these taxa). Vertebrates and plants had intermediate levels of focus (14.3% and 17.9%, respectively). Most respondents focused their research in the temperate areas (23.2%), while 89.3% indicated to have no checklist published in polar areas.

Respondents have published species checklists in 20 of the 22 United Nations geographical subregions (see Appendix S3), with particular focus in Southern Europe (26.8%), followed by South America (21.4%) and Western Europe (17.9%). We had no answers from experts who have published checklists in the Australia and New Zealand subregion and Polynesia subregion.

### 3.2 Reporting of non‐native species

**FIGURE 1:**
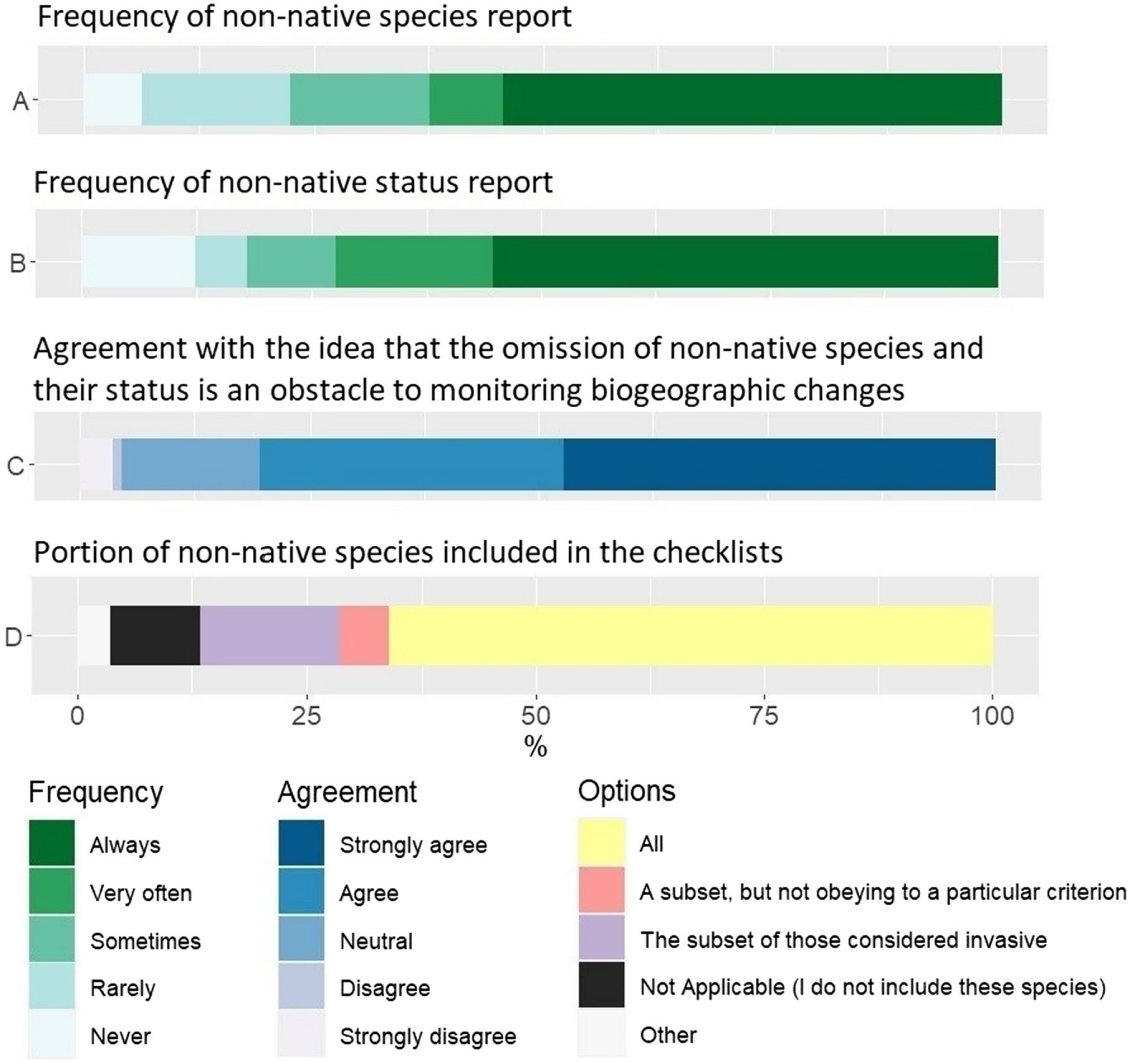
Summary of responses concerning reporting habits of non‐native species.

Of the 112 survey respondents, slightly less than half (45.5%) indicate that they do not always include non‐native established species in the checklists, with 6.3% (seven respondents) saying that they never include them (Figure 1A). When adding non‐native species to the checklists, most respondents mention that they list all they are aware of (66.1%), while 20.6% mention that they do not necessarily list all of them, with 15.2% mentioning to only report invasive species or subsets of these species that obey to no criterion (5.4%) (Figure 1D).

**FIGURE 2:**
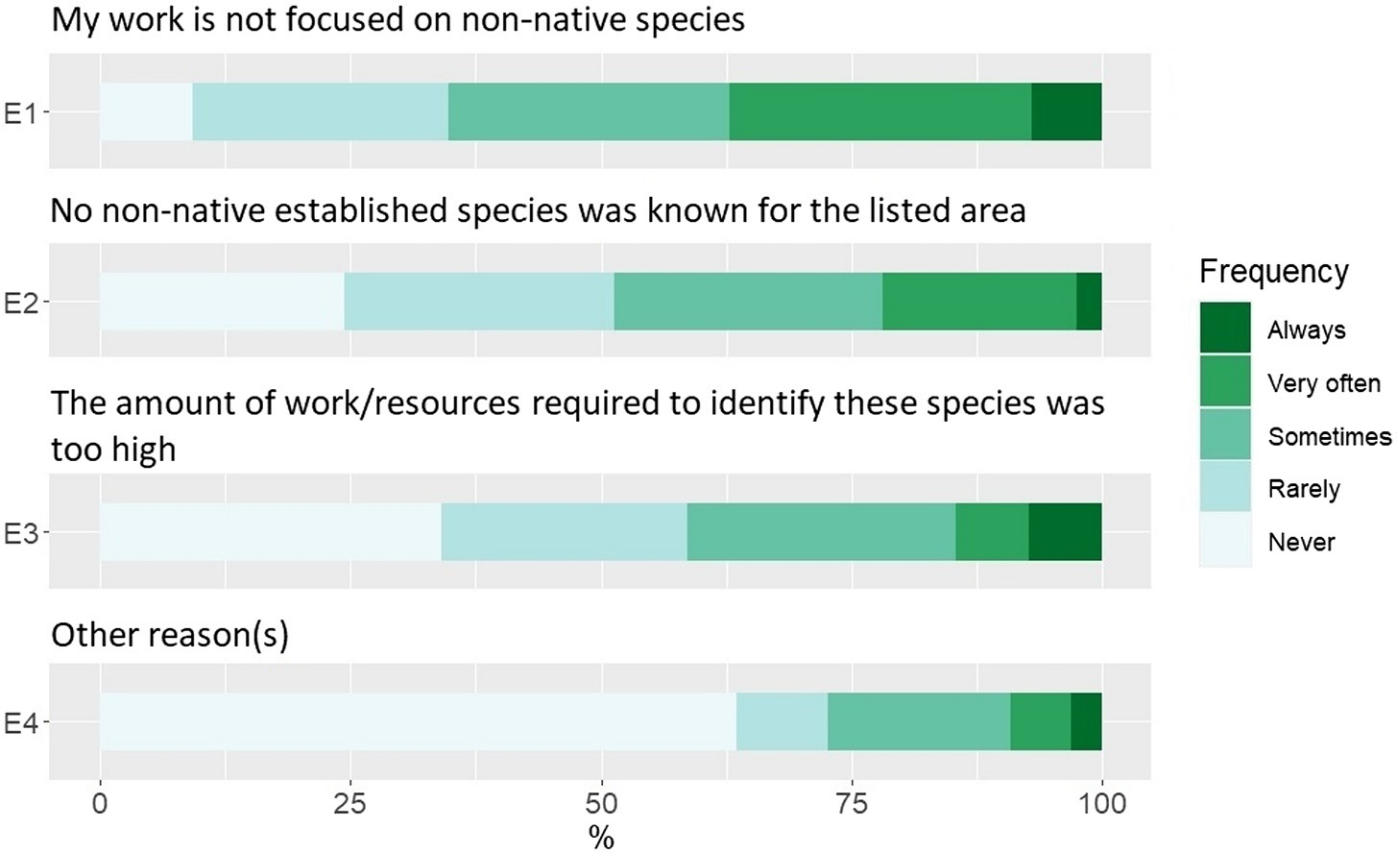
Summary of responses concerning reasons for not including non‐native species in checklists.

The reason most consistently pointed for not including non‐native species “always” is the additional amount of work or resources required (7.3%). However, when also considering causes mentioned to occur “sometimes” and “very often,” the most common reason becomes “my work is not focused on non‐native species” (58.1%) (Figure 2). Situations where no non‐native species are known in the region are also commonly pointed for their omission from the checklists, however, this ‘ideal’ situation corresponds to <10% of the cases indicated as occurring “always” and <25% when “very often” is also considered.

Concerning the reporting of the non‐native status of the species, 43.7% mention that they do not always provide this information (i.e., they do not differentiate native from non‐native species) and 18.1% of respondents mention that they never or rarely add this differentiation (Figure 1B). However, when asked about their agreement that the omission of established non‐native species from regional species lists or the non‐indication of their non‐native status is an obstacle to monitoring biogeographical and biodiversity change, most respondents agree or strongly agree (80.3%; Figure 1C).

### 3.3 Issues of terminology

**FIGURE 3:**
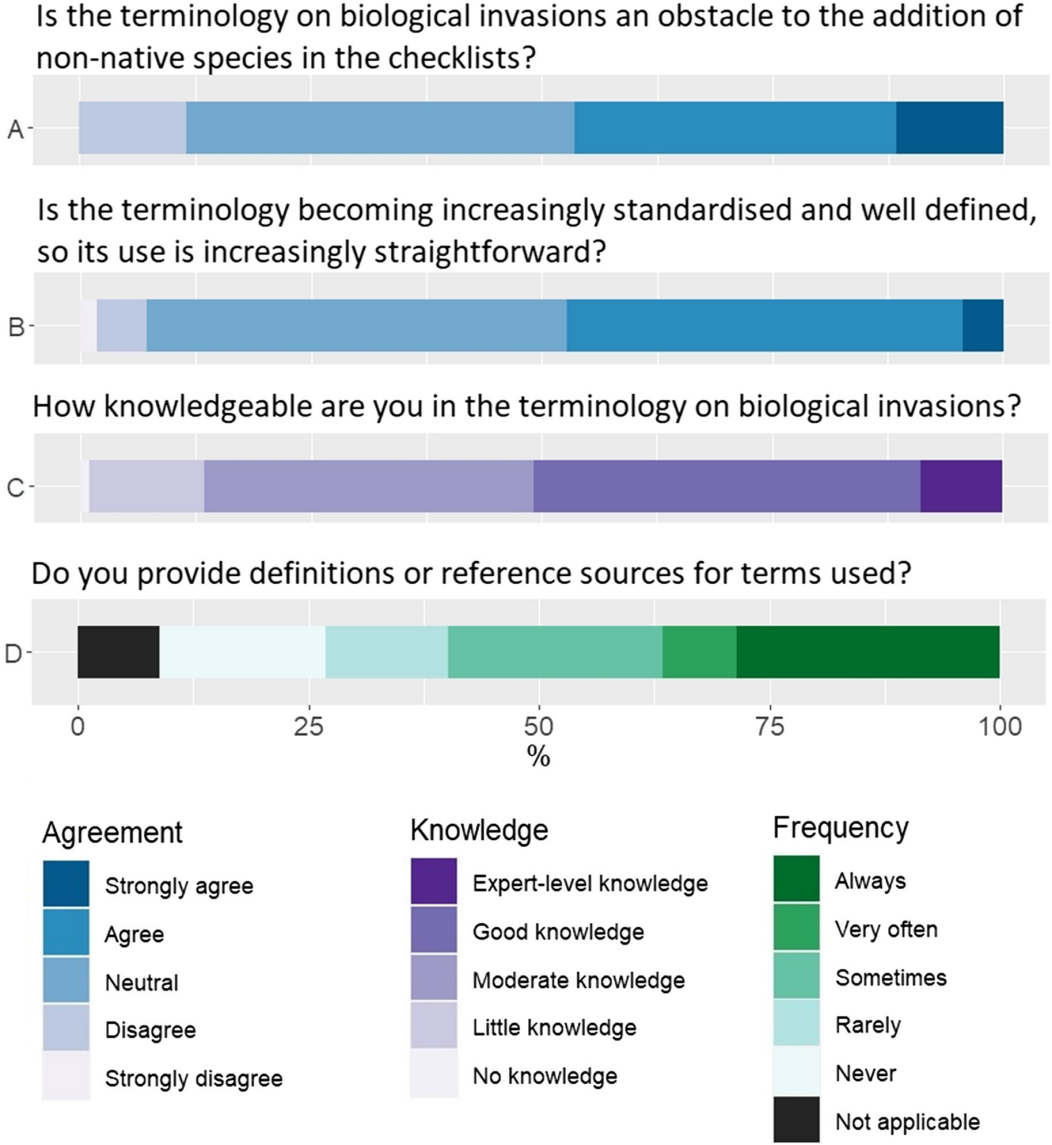
Summary of responses concerning issues of biological invasions terminology.

Concerning issues related to terminology, we found that nearly half (46.4%) of respondents agree or strongly agree that the terminology of biological invasions is an obstacle to the addition of non‐native established species in regional species checklists. A total of 42% respondents has no opinion about this and 11.6% disagree or strongly disagree (Figure 3A). Nonetheless, 47.4% agree or strongly agree that terminology on biological invasions and non‐native species is becoming increasingly standardized and well‐defined, so that the use of terms is increasingly straightforward. Most of the remaining respondents had a neutral perspective and only a few disagree with this assertion (Figure 3B). Concerning the knowledgeability of respondents on terms and definitions used in biological invasions, nearly 50% indicate to have no knowledge to moderate knowledge (Figure 3C). Finally, of those who include non‐native species in the checklists and mention them as such, the provision of a definition for used terms or the indication of a bibliographic reference is not always provided, with 31.3% never or rarely providing it, more than those who always provide it (28.6%; Figure 3D).

### 3.4 Data delivery

**FIGURE 4:**
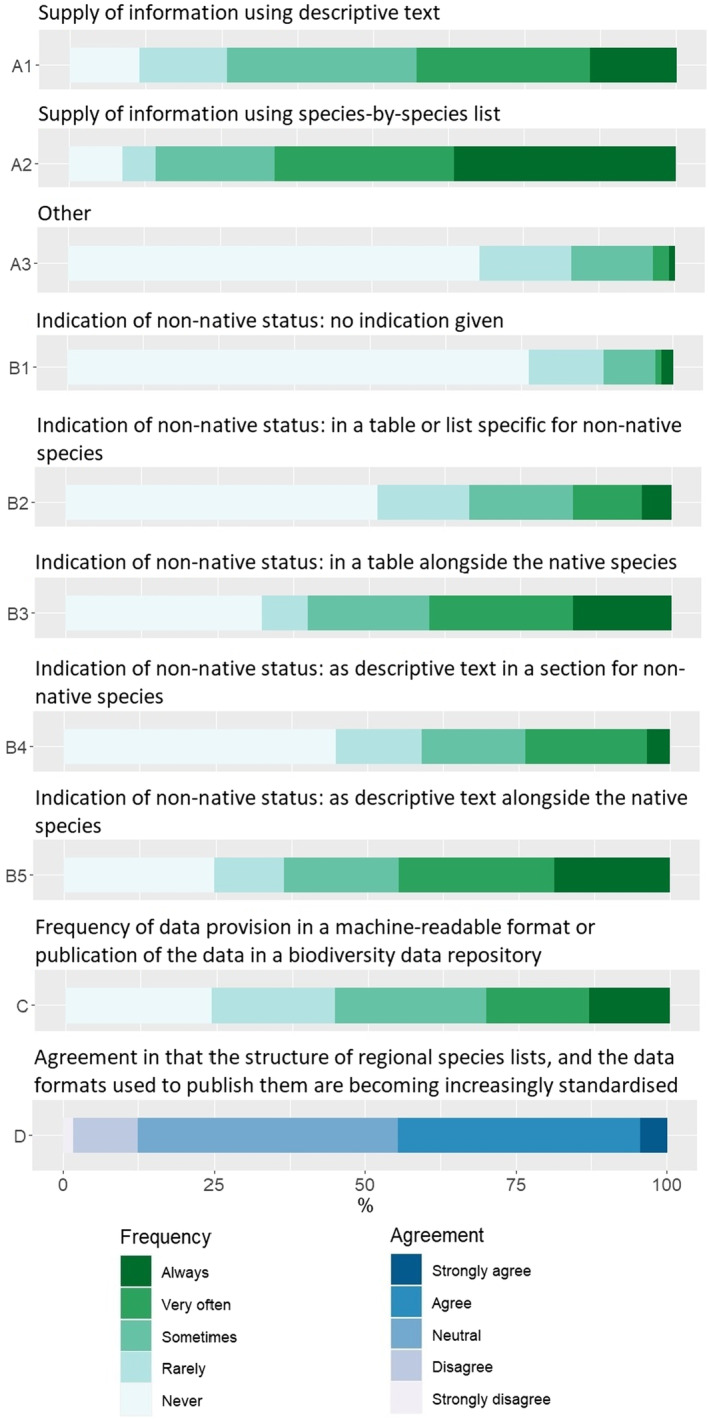
Summary of responses concerning data delivery.

Two‐thirds of respondents indicate that the listing of species is provided as a standardized table or list, summarizing the same categories of information for all species (Figure 4A2), whereas 42.9% mention that they provide a descriptive text for each species, either solely or as complement to a standardized table or list (Figure 4A1). A total of 44.7% of respondents who include non‐native species in the checklists, report the species' non‐native status using a descriptive text alongside the native species (“always” and “very often” answers). The second most used report option is tables alongside the native species (e.g., using symbols or other indications), with 40% of “very often” and “always” answers (Figure 4B3,B5).

Concerning the provisioning of the data in machine‐readable formats (e.g., excel file, CSV, or XML), only 13.4% of experts mention that they always provide these or publish the data in a standardized biodiversity data repository. Moreover, 44.6% mention that they never or rarely do this (Figure 4C). Nonetheless, 44.7% strongly agree or agree with the idea that the structure of regional species lists, and the data formats used to publish them are becoming increasingly standardized, with 42.9% having no opinion about it (Figure 4D).

### 3.5 Regressions analyses

**FIGURE 5:**
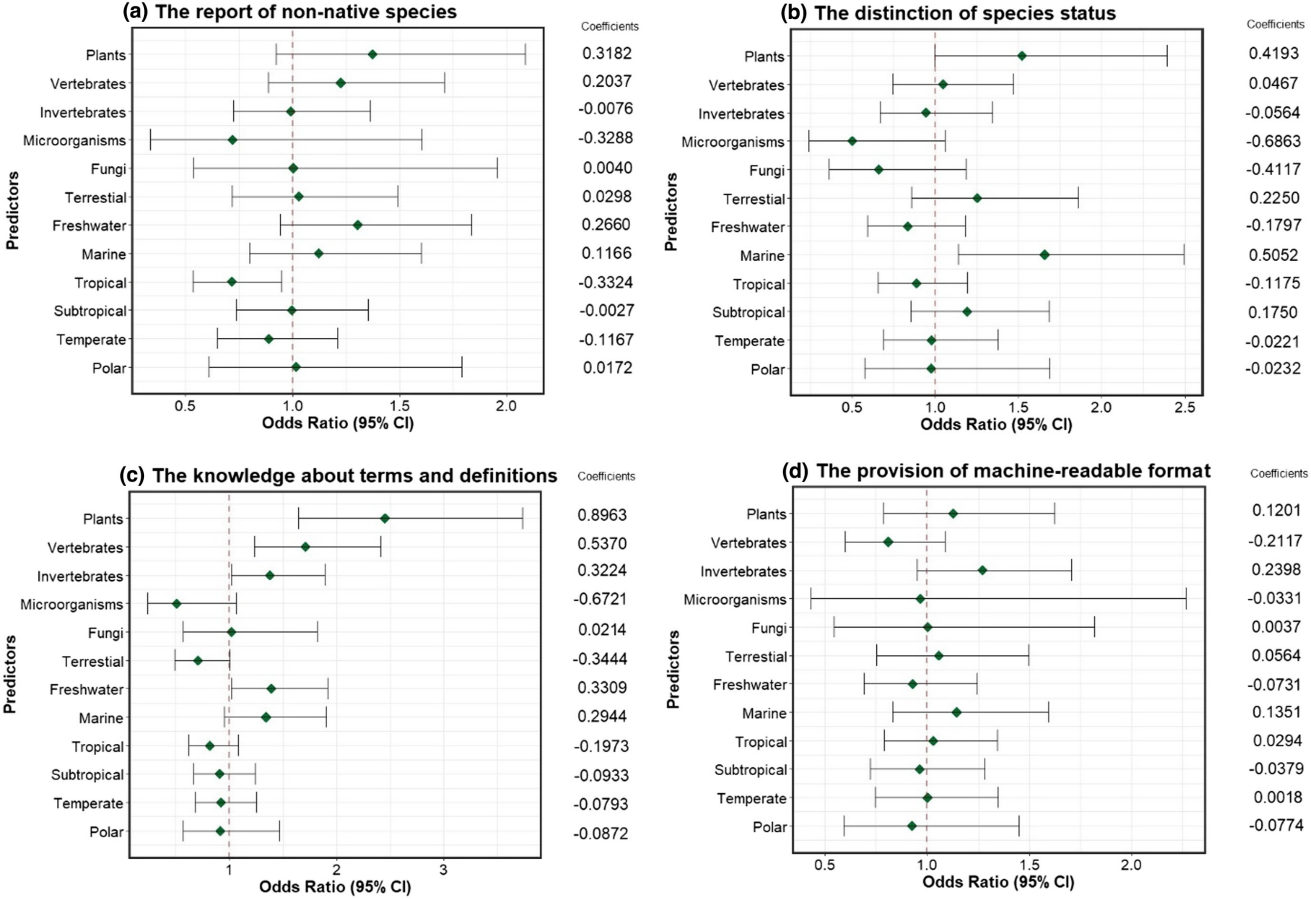
Results of ordinal logistic regressions, relating patterns of variation in answers and levels of expertise in distinct taxonomic groups, environmental realms and quantity of checklists published in each biome. The responses concern (a) the frequency of inclusion of non‐native species in the checklists; (b) the identification of non‐native species status; (c) knowledge about terms and definitions of biological invasions; (d) the provision of data in machine‐readable formats or publication in biodiversity data repositories. (See Appendix S4).

Ordinal logistic regression identified a significant (*α* = 0.05) negative relationship between the quantity of checklists published for tropical biomes and the frequency of reporting of non‐native species (Figure 5a). No significant relationships were found between this frequency and taxonomic or environmental realm expertise. When non‐native species are included in the checklists, the reporting of their non‐native status appears to be significantly more common among experts working in the marine realm (Figure 5b). Respondents with higher expertise in plants, vertebrates, invertebrates, and the freshwater environmental realm have a significant positive relationship with higher knowledge of the terms and definitions of biological invasions (Figure 5c). No significant relationship was found between respondents' expertise and research focus on distinct biomes, and the frequency of provision of data in machine‐readable formats (Figure 5d).

## 4 DISCUSSION

This work provides a detailed assessment of the attitudes and practices of non‐native species reporting by authors of regional species checklists. We found that nearly half of these authors do not always include non‐native taxa in the checklists, and when included their non‐native status is often omitted. Also close to half of the authors mention to have no to moderate‐only knowledge about the terms and definitions used in biological invasions and see this terminology as an obstacle to the inclusion of non‐native species in the checklists. Finally, checklists' data are dominantly published by means of unstructured data types and formats, such as free text and embedded tables in a pdf document, and only rarely are published in biodiversity data repositories.

The high frequency of omission of non‐native species from regional checklists is surprising and takes place despite most checklist authors agreeing that it hinders the monitoring of biogeographical and biodiversity change. Moreover, this omission appears to occur largely irrespectively of the taxonomic groups, environmental realms, and biomes of focus of the authors. The only exception to this concerns authors with a greater focus on tropical biomes, who tend to omit non‐native species more frequently. This relationship may be explained by a primary focus by these authors on listing only native taxa and discovering new species, as these areas are expected to retain the vast majority of terrestrial species that remain undescribed (Giam et al., 2012). In fact, a relevant number of checklist authors have indicated that the omission of non‐native taxa is simply due to the focus of their work not including these taxa. Another reason often given is the amount of additional resources that would be required to include these taxa, a factor that may be even more pressing in tropical areas, where pre‐existing levels of biodiversity information are generally lower and the resources needed for undertaking new field surveys are expectedly higher. Intriguingly, we found no relationship between the frequency of non‐native species reporting and level of research focus on the temperate regions. Arguably, a greater focus on these areas should be positively related to the propensity to report non‐native taxa, as temperate regions host the greatest richness of non‐native taxa (Dawson et al., 2017) and ratios of non‐native to native biodiversity (Sax, 2001). At the same time, these areas also host most economically developed countries having greater resources for biodiversity monitoring (Pereira et al., 2010). One possibility for this may be that, despite their prominence in these regions, non‐native species are generally regarded as a “distinct” assemblage, even among ecologists, and are often reported in isolation from native species, even though they play relevant roles in biological communities and represent an important portion of local biodiversity (Schlaepfer, 2018). That is, although the reporting of non‐native species occurs, it is done in isolation from native species, making it difficult to obtain aggregated information about the dynamics and trends of biodiversity in these areas.

Relevantly, checklists that include non‐native taxa often omit their non‐native status, a practice that also occurs largely regardless of the background of the checklist authors. The one exception is experts working in marine environments, who have a significant propensity to differentiate between native and non‐native taxa on checklists. The reason for this relationship is unclear. However, despite the paucity of overall knowledge about marine invasions (Ojaveer et al., 2015), marine and coastal ecosystems have been severely impacted by a set of prominent invasive species, often also massively impacting human activities either positively (e.g., red king crab *Paralithodes camtchaticus*, a non‐native species which is an important source of income for Norwegian seafood industry, Lorentzen et al., 2018) or negatively (e.g., sea star *Asterias amurensis* which feeds commercially valuable species in southern Australia, Ross et al., 2002). One possibility is that the societal notoriety of some of these species may have contributed to an overall awareness about the relevance of reporting their distribution and of other non‐native species.

A relevant portion of our survey participants view biological invasion terminology as an obstacle to the addition of non‐native species to regional checklists. Terminology and associated definitions are a key facet of invasive species reporting (Golebie et al., 2022), but also a recognized source of challenges, ambiguities, and disagreements among invasion ecologists (e.g., Essl et al., 2018; Mcgeoch et al., 2012; Wilson, 2020), which can be understood in part by the youth of biological invasions as a scientific discipline and the underlying conceptual differences in fields that comprise invasion research, such as biogeography, conservation, ecology, or evolutionary biology (Heger et al., 2013). However, it is now more than 10 years since notable efforts to harmonize terms and definitions have been published (Blackburn et al., 2011; Richardson et al., 2011), and there is now a prevailing view that these terms and definitions are increasingly standardized and well‐defined among the research community (Golebie et al., 2022), a view that is only contradicted by a very small percentage of our survey participants. Thus, it seems likely that a harmonized use of invasion terminology will become increasingly prevalent in regional species checklists as time progresses. However, we also found that, for the time being, a large percentage of checklist authors still have limited knowledge of this terminology. For example, significant positive relationships between taxonomic expertise and levels of terminology knowledge were found for plants, vertebrates, and invertebrates, but not for fungi and microorganisms groups for which knowledge about invasions is difficult to obtain and remains limited (Litchman, 2010; Monteiro et al., 2022). Thus, efforts to improve knowledge of this terminology and its harmonized adoption among checklist authors seem justified. This could be performed, in part, by the publication of terminology guidelines aimed specifically at biogeographical data providers (such as authors of species checklists), similar to what has recently been done for other application areas such as policy and management (Essl et al., 2018).

Our results also show that data in checklists are dominantly being published using unstructured data types and are only rarely submitted to biodiversity data repositories. These practices are sub‐optimal for data discovery and integration into analytical workflows, often requiring the use of manual procedures (Reyserhove et al., 2020). Furthermore, the inhibitory effects of these practices are intensified by the increasing volume of scientific publications (Bornmann & Mutz, 2015), a trend that is also likely occurring for species checklists. Thus, although regional species checklists are a prime source of information on biodiversity status and trends, often constituting the most comprehensive source of information for an area, including for non‐native species, the use of this information can pose major analytical challenges, particularly if integration of information contained in multiple lists is to be achieved. The ideal way to resolve these difficulties is undoubtedly through increasing standardization of the data structures and formats used to provide and store the data, as is being already promoted by some journals publishing species checklists (e.g., Biodiversity Data Journal), as well as by the publishing of data in standardized data repositories such as GBIF (gbif.org). Despite the preferential obviousness of this path, widespread adoption of these practices is unlikely to be achieved in full or in the near future. In this context, procedures for the automatic identification and extraction of data from unstructured data sources, such as pdf versions of published articles, may assume significant relevance. The ability to process unstructured data has grown greatly in recent years, driven largely by new computational tools, such as natural language processing models, capable of interpreting written text and extracting relevant information (Farrell et al., 2022). To date, these technologies have not yet provided a fully functional means of extracting complex custom‐based information from ecological and biogeographic literature, but recent advances, such as identifying species names in publications (Jarić et al., 2020; Le Guillarme & Thuiller, 2022), suggest that this may soon become possible.

We investigated the attitudes and practices of reporting of non‐native species in regional species checklists. While constituting a prime source of biogeographical information, regional species checklists still pose a number of pressing difficulties for biodiversity change and non‐native species research, including the total or partial omission of non‐native taxa and of associated information (such as native status), uncertainty and ambiguity in the terms and definitions used to classify these species and the common provision of information in an unstructured manner, limiting data assimilation and integration into automated workflows. Despite these challenges, the prevailing view among authors is that the addition of non‐native taxa to the checklists is valuable for assessing ongoing biogeographical and biodiversity changes. Thus, the time seems ripe for raising awareness about the relevance of listing these taxa and the adoption of standardized terminology and data sharing practices.

## AUTHOR CONTRIBUTIONS

**Andry Castro:** Conceptualization (lead); data curation (lead); formal analysis (lead); investigation (lead); methodology (lead); resources (lead); software (lead); visualization (lead); writing – original draft (lead); writing – review and editing (lead). **Joana Ribeiro:** Data curation (supporting); formal analysis (supporting); investigation (supporting); methodology (supporting); visualization (supporting); writing – review and editing (supporting). **Luis Reino:** Data curation (supporting); formal analysis (supporting); investigation (supporting); methodology (supporting); visualization (supporting); writing – review and editing (supporting). **César Capinha:** Conceptualization (lead); data curation (supporting); formal analysis (supporting); investigation (lead); methodology (lead); resources (supporting); software (supporting); validation (lead); visualization (supporting); writing – original draft (lead); writing – review and editing (lead).

## FUNDING INFORMATION

AC was supported by a grant (PRT/BD/152100/2021) financed by the Portuguese Foundation for Science and Technology (FCT) under MIT Portugal Program. AC and CC acknowledge support from FCT through support to CEG/IGOT Research Unit (UIDB/00295/2020 and UIDP/00295/2020). JR was funded through project UNRAVEL (PTDC/BIA‐ECO/0207/2020), financed by National Funds through the FCT. LR was supported by Portuguese National Funds through FCT, public institute (IP), through project UNRAVEL (PTDC/BIA‐ECO/0207/2020) and under the Stimulus of Scientific Employment: Individual Support contract no. CEECIND/00445/2017.

## CONFLICT OF INTEREST STATEMENT

No conflict of interest is declared.

## Supporting information


Appendix S1‐S4

Appendix S2

## Data Availability

The data used in this study include human subject data that cannot be shared publicly.

## References

[ece310148-bib-0001] Bezerra, C. H. , Pinheiro, L. T. , de Melo, G. C. , Zanchi‐Silva, D. , Queiroz Mde, S. , dos Anjos, L. , Harris, D. J. , & Borges‐Nojosa, D. M. (2016). Assessing the influence of geographic distance in parasite communities of an exotic lizard. Acta Parasitologica, 61(1), 136–143. 10.1515/ap-2016-0018 26751884

[ece310148-bib-0002] Blackburn, T. M. , Pyšek, P. , Bacher, S. , Carlton, J. T. , Duncan, R. P. , Jarošík, V. , Wilson, J. R. , & Richardson, D. M. (2011). A proposed unified framework for biological invasions. Trends in Ecology and Evolution, 26(7), 333–339. 10.1016/j.tree.2011.03.023 21601306

[ece310148-bib-0003] Bornmann, L. , & Mutz, R. (2015). Growth rates of modern science: A bibliometric analysis based on the number of publications and cited references. Journal of the Association for Information Science and Technology, 66(11), 2215–2222. 10.1002/asi.23329

[ece310148-bib-0004] Brondízio, E. S. , Settele, J. , Díaz, S. , & Ngo, H. T. (2019). The global assessment report on biodiversity and ecosystem services. IPBES secretariat.

[ece310148-bib-0005] Capinha, C. , Marcolin, F. , & Reino, L. (2020). Human‐induced globalization of insular herpetofaunas. Global Ecology and Biogeography, 29(8), 1328–1349. 10.1111/geb.13109

[ece310148-bib-0006] Chase, J. M. , McGill, B. J. , Thompson, P. L. , Antão, L. H. , Bates, A. E. , Blowes, S. A. , Dornelas, M. , Gonzalez, A. , Magurran, A. E. , Supp, S. R. , Winter, M. , Bjorkman, A. D. , Bruelheide, H. , Byrnes, J. E. K. , Cabral, J. S. , Elahi, R. , Gomez, C. , Guzman, H. M. , Isbell, F. , … O'Connor, M. (2019). Species richness change across spatial scales. Oikos, 128(8), 1079–1091. 10.1111/oik.05968

[ece310148-bib-0007] Collen, B. , Ram, M. , Zamin, T. , & McRae, L. (2008). The tropical biodiversity data gap: Addressing disparity in global monitoring. Tropical Conservation Science, 1(2), 75–88. 10.1177/194008290800100202

[ece310148-bib-0008] Dawson, W. , Moser, D. , Van Kleunen, M. , Kreft, H. , Pergl, J. , Pyšek, P. , Weigelt, P. , Winter, M. , Lenzner, B. , Blackburn, T. M. , Dyer, E. E. , Cassey, P. , Scrivens, S. L. , Economo, E. P. , Guénard, B. , Capinha, C. , Seebens, H. , García‐Díaz, P. , Nentwig, W. , … Essl, F. (2017). Global hotspots and correlates of alien species richness across taxonomic groups. Nature Ecology Evolution, 1, 1–7. 10.1038/s41559-017-0186 28812620

[ece310148-bib-0009] Essl, F. , Bacher, S. , Genovesi, P. , Hulme, P. E. , Jeschke, J. M. , Katsanevakis, S. , Kowarik, I. , Kühn, I. , Pyšek, P. , Rabitsch, W. , Schindler, S. , van Kleunen, M. , Vilà, M. , Wilson, J. R. U. , & Richardson, D. M. (2018). Which taxa are alien? Criteria, applications, and uncertainties. Bioscience, 68(7), 496–509. 10.1093/biosci/biy057

[ece310148-bib-0010] Farrell, M. J. , Brierley, L. , Willoughby, A. , Yates, A. , & Mideo, N. (2022). Past and future uses of text mining in ecology and evolution. Proceedings of the Royal Society B: Biological Sciences, 289(1975), 1–9. 10.1098/rspb.2021.2721 PMC9114983 35582795

[ece310148-bib-0011] Gallardo, B. , Clavero, M. , Sánchez, M. I. , & Vilà, M. (2016). Global ecological impacts of invasive species in aquatic ecosystems. Global Change Biology, 22(1), 151–163. 10.1111/gcb.13004607 26212892

[ece310148-bib-0012] Giam, X. , Scheffers, B. R. , Sodhi, N. S. , Wilcove, D. S. , Ceballos, G. , & Ehrlich, P. R. (2012). Reservoirs of richness: Least disturbed tropical forests are centres of undescribed species diversity. Proceedings of the Royal Society B: Biological Sciences, 279(1726), 67–76. 10.1098/rspb.2011.0433 PMC3223638 21593037

[ece310148-bib-0013] Golebie, E. J. , van Riper, C. J. , Arlinghaus, R. , Gaddy, M. , Jang, S. , Kochalski, S. , Olden, J. D. , Stedman, R. C. , & Suski, C. (2022). Words matter: A systematic review of communication in non‐native aquatic species literature. NeoBiota, 74, 1–28. 10.3897/neobiota.74.79942

[ece310148-bib-0014] Heger, T. , Saul, W. C. , & Trepl, L. (2013). What biological invasions ‘are’ is a matter of perspective. Journal for Nature Conservation, 21(2), 93–96. 10.1016/j.jnc.2012.11.002

[ece310148-bib-0015] Jarić, I. , Correia, R. A. , Brook, B. W. , Buettel, J. C. , Courchamp, F. , di Minin, E. , Firth, J. A. , Gaston, K. J. , Jepson, P. , Kalinkat, G. , Ladle, R. , Soriano‐Redondo, A. , Souza, A. T. , & Roll, U. (2020). iEcology: Harnessing large online resources to generate ecological insights. Trends in Ecology and Evolution, 35(7), 630–639. 10.1016/j.tree.2020.03.003 32521246

[ece310148-bib-0016] Le Guillarme, N. , & Thuiller, W. (2022). TaxoNERD: Deep neural models for the recognition of taxonomic entities in the ecological and evolutionary literature. Methods in Ecology and Evolution, 13(3), 625–641. 10.1111/2041-210X.13778

[ece310148-bib-0017] Likert, R. (1932). A technique for the measurement of attitudes. Archives of Psychology.

[ece310148-bib-0018] Litchman, E. (2010). Invisible invaders: Non‐pathogenic invasive microbes in aquatic and terrestrial ecosystems. Ecology Letters, 13(12), 1560–1572. 10.1111/j.1461-0248.2010.01544.x 21054733

[ece310148-bib-0019] Lorentzen, G. , Voldnes, G. , Whitaker, R. D. , Kvalvik, I. , Vang, B. , Gjerp Solstad, R. , Thomassen, M. R. , & Siikavuopio, S. I. (2018). Current status of the red king crab (*Paralithodes camtchaticus*) and snow crab (*Chionoecetes opilio*) industries in Norway. Reviews in Fisheries Science and Aquaculture, 26(1), 42–54. 10.1080/23308249.2017.1335284

[ece310148-bib-0021] McGeoch, M. A. , Spear, D. , Kleynhans, E. J. , & Marais, E. (2012). Uncertainty in invasive alien species listing. Ecological Applications, 22(3), 959–971. 10.2307/23213930 22645824

[ece310148-bib-0022] McGeoch, M. A. , Butchart, S. H. M. , Spear, D. , Marais, E. , Kleynhans, E. J. , Symes, A. , Chanson, J. , & Hoffmann, M. (2010). Global indicators of biological invasion: Species numbers, biodiversity impact and policy responses. Diversity and Distributions, 16(1), 95–108. 10.1111/j.1472-4642.2009.00633.x

[ece310148-bib-0023] Monteiro, M. , Reino, L. , Ferreira, M. T. , Schertler, A. , & Capinha, C. (2022). Patterns and drivers of the global diversity of non‐native macrofungi. Diversity and Distributions, 28, 2042–2055. 10.1111/ddi.13

[ece310148-bib-0024] Ojaveer, H. , Galil, B. S. , Campbell, M. L. , Carlton, J. T. , Canning‐Clode, J. , Cook, E. J. , Davidson, A. D. , Hewitt, C. L. , Jelmert, A. , Marchini, A. , McKenzie, C. , Minchin, D. , Occhipinti‐Ambrogi, A. , Olenin, S. , & Ruiz, G. (2015). Classification of non‐indigenous species based on their impacts: Considerations for application in marine management. PLoS Biology, 13(4), 1–13. 10.1371/journal.pbio.1002130 PMC4398364 25875845

[ece310148-bib-0025] Pereira, H. M. , Belnap, J. , Brummitt, N. , Collen, B. , Ding, H. , Gonzalez‐Espinosa, M. , Gregory, R. D. , Honrado, J. , Jongman, R. H. G. , Julliard, R. , McRae, L. , Proenca, V. , Rodrigues, P. , & Vieira, C. (2010). Global biodiversity monitoring. Frontiers in Ecology and the Environment, 8(9), 459–460. 10.2307/29546158

[ece310148-bib-0026] Pyšek, P. , Hulme, P. E. , Simberloff, D. , Bacher, S. , Blackburn, T. M. , Carlton, J. T. , Dawson, W. , Essl, F. , Foxcroft, L. C. , Genovesi, P. , Jeschke, J. M. , Kühn, I. , Liebhold, A. M. , Mandrak, N. E. , Meyerson, L. A. , Pauchard, A. , Pergl, J. , Roy, H. E. , Seebens, H. , … Richardson, D. M. (2020). Scientists' warning on invasive alien species. Biological Reviews, 95(6), 1511–1534. 10.1111/brv.12627 32588508 PMC7687187

[ece310148-bib-0027] Pyšek, P. , Richardson, D. M. , Rejmánek, M. , Webster, G. L. , Williamson, M. , & Kirschner, J. (2004). Alien plants in checklists and floras: Towards better communication between taxonomists and ecologists. Taxon, 53(1), 131–143. 10.2307/4135498

[ece310148-bib-0028] R Core Team . (2022). R: A language and environment for statistical computing. R Foundation for Statistical Computing.

[ece310148-bib-0029] Reyserhove, L. , Desmet, P. , Oldoni, D. , Adriaens, T. , Strubbe, D. , Davis, A. J. S. , Vanderhoeven, S. , Verloove, F. , & Groom, Q. (2020). A checklist recipe: Making species data open and FAIR. Database, 2020(1), 1–12. 10.1093/database/baaa084 33181821 PMC7661093

[ece310148-bib-0030] Ribeiro, J. , Reino, L. , Schindler, S. , Strubbe, D. , Vall‐llosera, M. , Araújo, M. B. , Capinha, C. , Carrete, M. , Mazzoni, S. , Monteiro, M. , Moreira, F. , Rocha, R. , Tella, J. L. , Vaz, A. S. , Vicente, J. , & Nuno, A. (2019). Trends in legal and illegal trade of wild birds: A global assessment based on expert knowledge. Biodiversity and Conservation, 28(12), 3343–3369. 10.1007/s10531-019-01825-5

[ece310148-bib-0031] Richardson, D. M. , Pyšek, P. , & Carlton, J. T. (2011). A compendium of essential concepts and terminology in invasion ecology. In Fifty years of invasion ecology: The legacy of Charles Elton (pp. 409–420). Wiley‐Blackwell. 10.1002/9781444329988.ch30

[ece310148-bib-0032] Ross, D. J. , Johnson, C. R. , & Hewitt, C. L. (2002). Impact of introduced seastars *Asterias amurensis* on survivorship of juvenile commercial bivalves *Fulvia tenuicostata* . Marine Ecology Progress Series, 241, 99–112. 10.3354/meps241099

[ece310148-bib-0033] Sala, O. E. , Chapin, F. S. , Armesto, J. J. , Berlow, E. , Bloomfield, J. , Dirzo, R. , Huber‐Sanwald, E. , Huenneke, L. F. , Jackson, R. B. , Kinzig, A. , Leemans, R. , Lodge, D. M. , Mooney, H. A. , Oesterheld, M. , Poff, N. L. , Sykes, M. T. , Walker, B. H. , Walker, M. , & Wall, D. H. (2000). Global biodiversity scenarios for the year 2100. Science, 287(5459), 1770–1774. 10.1126/science.287.5459.1770 10710299

[ece310148-bib-0034] Sax, D. F. (2001). Latitudinal gradients and geographic ranges of exotic species: Implications for biogeography. Journal of Biogeography, 28(1), 139–150. 10.1046/j.1365-2699.2001.00536.x

[ece310148-bib-0035] Schlaepfer, M. A. (2018). Do non‐native species contribute to biodiversity? PLoS Biology, 16(4), 1–6. 10.1371/journal.pbio.2005568 PMC5903594 29664943

[ece310148-bib-0036] Seebens, H. , Blackburn, T. M. , Dyer, E. E. , Genovesi, P. , Hulme, P. E. , Jeschke, J. M. , Pagad, S. , Pyšek, P. , Winter, M. , Arianoutsou, M. , Bacher, S. , Blasius, B. , Brundu, G. , Capinha, C. , Celesti‐Grapow, L. , Dawson, W. , Dullinger, S. , Fuentes, N. , Jäger, H. , … Essl, F. (2017). No saturation in the accumulation of alien species worldwide. Nature Communications, 8(14435), 1–9. 10.1038/ncomms14435 PMC5316856 28198420

[ece310148-bib-0037] Seebens, H. , Clarke, D. A. , Groom, Q. , Wilson, J. R. U. , García‐Berthou, E. , Kühn, I. , Roigé, M. , Pagad, S. , Essl, F. , Vicente, J. , Winter, M. , & McGeoch, M. (2020). A workflow for standardising and integrating alien species distribution data. NeoBiota, 59, 39–59. 10.3897/NEOBIOTA.59.53578

[ece310148-bib-0038] Seebens, H. , & Kaplan, E. (2022). DASCO: A workflow to downscale alien species checklists using occurrence records and to re‐allocate species distributions across realms. NeoBiota, 74, 75–91. 10.3897/neobiota.74.81082

[ece310148-bib-0039] Shackleton, R. T. , Shackleton, C. M. , & Kull, C. A. (2019). The role of invasive alien species in shaping local livelihoods and human well‐being: A review. Journal of Environmental Management, 229, 145–157. 10.1016/j.jenvman.2018.05.007 30049620

[ece310148-bib-0040] Tiago, P. , Gouveia, M. J. , Capinha, C. , Santos‐Reis, M. , & Pereira, H. M. (2017). The influence of motivational factors on the frequency of participation in citizen science activities. Nature Conservation, 18, 61–78. 10.3897/natureconservation.18.13429

[ece310148-bib-0041] Troudet, J. , Grandcolas, P. , Blin, A. , Vignes‐Lebbe, R. , & Legendre, F. (2017). Taxonomic bias in biodiversity data and societal preferences. Scientific Reports, 7(9132), 1–14. 10.1038/s41598-017-09084-6 28831097 PMC5567328

[ece310148-bib-0042] Venables, W. N. , & Springer, B. D. R. (2002). Modern applied statistics with S (4th ed.). Springer.

[ece310148-bib-0043] Wardle, D. A. , & Peltzer, D. A. (2017). Impacts of invasive biota in forest ecosystems in an aboveground–belowground context. Biological Invasions, 19(11), 3301–3316. 10.1007/s10530-017-1372-x

[ece310148-bib-0044] Wieczorek, J. , Bloom, D. , Guralnick, R. , Blum, S. , Döring, M. , Giovanni, R. , Robertson, T. , & Vieglais, D. (2012). Darwin Core: An evolving community‐developed biodiversity data standard. PLoS One, 7(1), 1–8. 10.1371/journal.pone.0029715 PMC3253084 22238640

[ece310148-bib-0045] Wilson, J. R. U. (2020). Definitions can confuse: Why the ‘neonative' neologism is bad for conservation. Bioscience, 70(2), 110–111. 10.1093/biosci/biz159

[ece310148-bib-0046] Zenni, R. D. , Essl, F. , García‐Berthou, E. , & McDermott, S. M. (2021). The economic costs of biological invasions around the world. NeoBiota, 67, 1–9. 10.3897/neobiota.67.69971

