# Supplementary material for: Who is reporting non‐native species and how? A cross‐expert assessment of practices and drivers of non‐native biodiversity reporting in species regional listing
Source: Ecol Evol. 2023 May 28;13(5):e10148. doi: 10.1002/ece3.10148 (PMC10225815; doi:10.1002/ece3.10148)
Supplement: Supplementary file 1 — Appendix S1‐S4 [file ECE3-13-e10148-s002.docx]

**Supporting Information**

**Appendix S1**


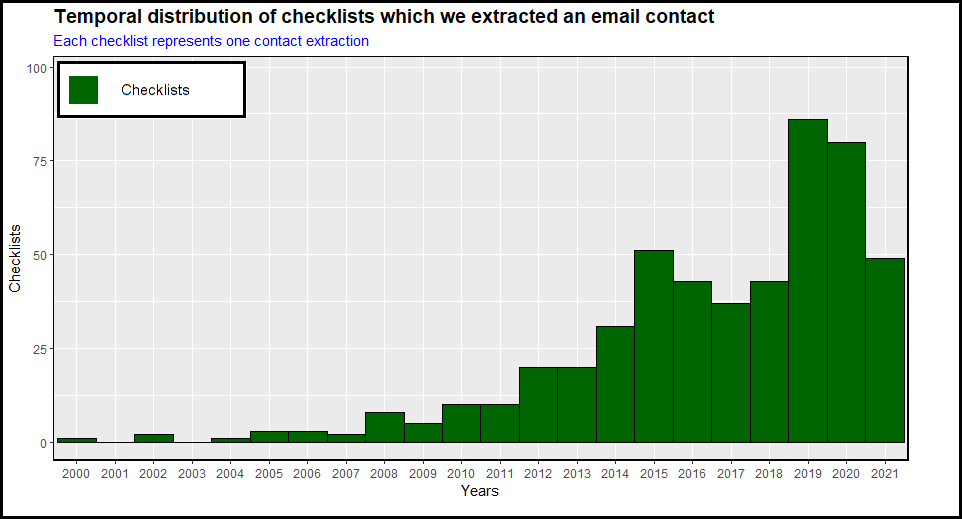


**Fig. S1.1 –** Temporal distribution of checklists which we extracted an email contact to invite experts to participate in the survey

**Appendix S2 –** Online survey

**Appendix S3**
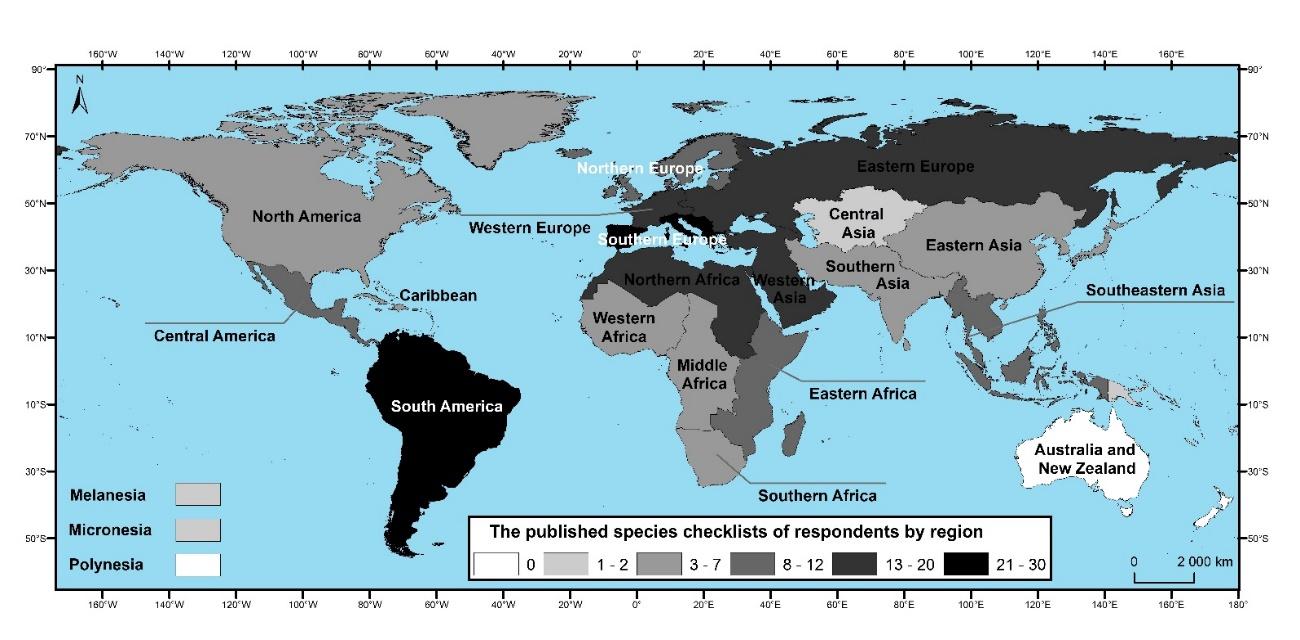


**Fig. S3.1 -** Geographical distribution of survey respondents. Grey shades represent the number respondents who have published species checklists in the respective subregion. Subregions are delineated according to United Nations geographical scheme

**Appendix S4**

**Table. S4.1 – Results of ordinal logistic regressions (The report of non-native species)**

|  | | | | |  |  |
| --- | --- | --- | --- | --- | --- | --- |
| Preditor | Coefficient | Std Error | *p* value | odds ratio | CI 2,5% | CI 97,5% |
| Taxonomic groups |  |  |  |  |  |  |
| Plants | 0,3182 | 0,2073 | 0,1247 | 1,3747 | 0,923 | 2,089 |
| Vertebrates | 0,2037 | 0,1665 | 0,2213 | 1,2259 | 0,886 | 1,708 |
| Invertebrates | -0,0076 | 0,1602 | 0,9619 | 0,9924 | 0,724 | 1,364 |
| Microorganisms | -0,3288 | 0,3929 | 0,4028 | 0,7198 | 0,336 | 1,604 |
| Fungi | 0,0040 | 0,3247 | 0,9902 | 1,0040 | 0,538 | 1,958 |
| Environmental realms |  |  |  |  |  |  |
| Terrestial | 0,0298 | 0,1850 | 0,8722 | 1,0302 | 0,719 | 1,492 |
| Freshwater | 0,2660 | 0,1687 | 0,1149 | 1,3047 | 0,944 | 1,836 |
| Marine | 0,1166 | 0,1760 | 0,5076 | 1,1237 | 0,799 | 1,602 |
| Biomes |  |  |  |  |  |  |
| Tropical | -0,3324 | 0,1457 | 0,0225 | 0,7172 | 0,534 | 0,950 |
| Subtropical | -0,0027 | 0,1537 | 0,9859 | 0,9973 | 0,739 | 1,355 |
| Temperate | -0,1167 | 0,1585 | 0,4615 | 0,8898 | 0,648 | 1,211 |
| Polar | 0,0172 | 0,2666 | 0,9486 | 1,0174 | 0,610 | 1,792 |

**Table. S4.2 – Results of ordinal logistic regressions (The distinction of species status)**

|  | | | | |  |  |
| --- | --- | --- | --- | --- | --- | --- |
| Preditor | Coefficient | Std Error | *p* value | odds ratio | CI 2,5% | CI 97,5% |
| Taxonomic groups |  |  |  |  |  |  |
| Plants | 0,4193 | 0,2223 | 0,0593 | 1,5209 | 0,9944 | 2,3900 |
| Vertebrates | 0,0467 | 0,1718 | 0,7857 | 1,0478 | 0,7467 | 1,4706 |
| Invertebrates | -0,0564 | 0,1773 | 0,7503 | 0,9451 | 0,6676 | 1,3445 |
| Microorganisms | -0,6863 | 0,3705 | 0,0640 | 0,5034 | 0,2413 | 1,0575 |
| Fungi | -0,4117 | 0,2989 | 0,1684 | 0,6625 | 0,3622 | 1,1846 |
| Environmental realms |  |  |  |  |  |  |
| Terrestial | 0,2250 | 0,1958 | 0,2506 | 1,2523 | 0,8582 | 1,8582 |
| Freshwater | -0,1797 | 0,1749 | 0,3043 | 0,8355 | 0,5922 | 1,1811 |
| Marine | 0,5052 | 0,1979 | 0,0107 | 1,6573 | 1,1407 | 2,4936 |
| Biomes |  |  |  |  |  |  |
| Tropical | -0,1175 | 0,1520 | 0,4396 | 0,8892 | 0,6557 | 1,1952 |
| Subtropical | 0,1750 | 0,1726 | 0,3106 | 1,1913 | 0,8521 | 1,6838 |
| Temperate | -0,0221 | 0,1746 | 0,8993 | 0,9781 | 0,6908 | 1,3766 |
| Polar | -0,0232 | 0,2668 | 0,9308 | 0,9771 | 0,5778 | 1,6864 |

**Table. S4.3 – Results of ordinal logistic regressions (The knowledge about terms and definitions)**

|  | | | | | |  |
| --- | --- | --- | --- | --- | --- | --- |
| Preditor | Coefficient | Std Error | *p* value | odds ratio | CI 2,5% | CI 97,5% |
| Taxonomic groups |  |  |  |  |  |  |
| Plants | 0,8963 | 0,2081 | 0,00002 | 2,4506 | 1,6458 | 3,7337 |
| Vertebrates | 0,5370 | 0,1705 | 0,002 | 1,7109 | 1,2320 | 2,4094 |
| Invertebrates | 0,3224 | 0,1579 | 0,041 | 1,3805 | 1,0166 | 1,8929 |
| Microorganisms | -0,6721 | 0,3768 | 0,074 | 0,5107 | 0,2383 | 1,0632 |
| Fungi | 0,0214 | 0,2939 | 0,942 | 1,0216 | 0,5694 | 1,8187 |
| Environmental realms |  |  |  |  |  |  |
| Terrestial | -0,3444 | 0,1806 | 0,056 | 0,7087 | 0,4942 | 1,0054 |
| Freshwater | 0,3309 | 0,1606 | 0,039 | 1,3922 | 1,0202 | 1,9189 |
| Marine | 0,2944 | 0,1759 | 0,094 | 1,3423 | 0,9531 | 1,9057 |
| Biomes |  |  |  |  |  |  |
| Tropical | -0,1973 | 0,1414 | 0,163 | 0,8209 | 0,6201 | 1,0817 |
| Subtropical | -0,0933 | 0,1565 | 0,551 | 0,9109 | 0,6692 | 1,2384 |
| Temperate | -0,0793 | 0,1559 | 0,611 | 0,9237 | 0,6790 | 1,2536 |
| Polar | -0,0872 | 0,2380 | 0,714 | 0,9165 | 0,5726 | 1,4694 |

**Table. S4.4 – Results of ordinal logistic regressions (The provision of machine-readble format)**

|  | | | | | |  |
| --- | --- | --- | --- | --- | --- | --- |
| Preditor | Coefficient | Std Error | *p* value | odds ratio | CI 2,5% | CI 97,5% |
| Taxonomic groups |  |  |  |  |  |  |
| Plants | 0,1201 | 0,1845 | 0,5150 | 1,1277 | 0,7863 | 1,6252 |
| Vertebrates | -0,2117 | 0,1522 | 0,1643 | 0,8092 | 0,5982 | 1,0884 |
| Invertebrates | 0,2398 | 0,1487 | 0,1067 | 1,2710 | 0,9511 | 1,7074 |
| Microorganisms | -0,0331 | 0,4185 | 0,9369 | 0,9674 | 0,4312 | 2,2671 |
| Fungi | 0,0037 | 0,3048 | 0,9904 | 1,0037 | 0,5447 | 1,8199 |
| Environmental realms |  |  |  |  |  |  |
| Terrestial | 0,0564 | 0,1738 | 0,7455 | 1,0580 | 0,7541 | 1,4980 |
| Freshwater | -0,0731 | 0,1496 | 0,6252 | 0,9295 | 0,6913 | 1,2452 |
| Marine | 0,1351 | 0,1648 | 0,4121 | 1,1447 | 0,8319 | 1,5926 |
| Biomes |  |  |  |  |  |  |
| Tropical | 0,0294 | 0,1345 | 0,8267 | 1,0299 | 0,7911 | 1,3436 |
| Subtropical | -0,0379 | 0,1463 | 0,7958 | 0,9629 | 0,7224 | 1,2839 |
| Temperate | 0,0018 | 0,1503 | 0,9905 | 1,0018 | 0,7459 | 1,3467 |
| Polar | -0,0774 | 0,2259 | 0,7318 | 0,9255 | 0,5920 | 1,4512 |
