# Supplementary material for: Who is reporting non‐native species and how? A cross‐expert assessment of practices and drivers of non‐native biodiversity reporting in species regional listing
Source: Ecol Evol. 2023 May 28;13(5):e10148. doi: 10.1002/ece3.10148 (PMC10225815; doi:10.1002/ece3.10148)
Supplement: Supplementary file 2 — Appendix S2 [file ECE3-13-e10148-s001.pdf]

Enhancing the potential of checklists for non-native species monitoring

Dear Participant,
Regional species checklists (defined as any publication that provides a list of species known to occur in a region, site, etc.) are an invaluable tool for mapping and monitoring the distribution of species. The relevance and potential of these publications for monitoring the distribution of non-native species is also high, but several issues continue to hinder their full realisation for this purpose. Since you are part of the scientific community that has published species checklists in recent years, I invite you to participate in this survey which aims to assess the attitudes and problems experienced by authors of regional species checklists towards non-native taxa. The survey will require approximately 10 minutes to complete.

The data collected will be kept for a maximum period of 5 years after the publication of the study. The information you provide is anonymous and the questions do not allow personal identification. This information will be analysed later and will only be accessible to the researchers of this study. The processing of the information we will carry out will be exclusively for scientific purposes. If you wish to receive the results of this study, given that the study is anonymous, please contact us through the email provided.

By starting to complete this questionnaire, you indicate that you have read and understood the above information and agree to read all the information carefully and answer all questions honestly.

We thank you in advance for your time. This data collection has been approved by IGOT-ULisboa ethics committee.

Sincerely,
Andry Castro (andrycastro@edu.ulisboa.pt)
Institute of Geography and Spatial Planning, University of Lisbon (IGOT-UL/CEG-UL)

César Capinha (Supervisor)
Institute of Geography and Spatial Planning, University of Lisbon (IGOT/CEG-UL)

João Pinto (Co-Supervisor)
Institute of Hygiene and Tropical Medicine (IHMT-UNL)

*Required

1. Please confirm that you understood the conditions to fill this questionnaire? (If you answer "no" to this question, the questionnaire ends here) *

   Mark only one oval.

   ◯ Yes

   ◯ No

Section A - Expertise confirmation

Below you will find definitions that you should consider in your answers.

Regional species checklist (also known as regional species list or inventory): refers to any type of publication (scientific article; dataset; technical report, etc.) that provides a list of all, or part of, the species known to occur in a region, such as a country, sub-national administrative division, or other location (e.g., a wetland; farm; protected area, etc.).

Established non-native species: a species introduced intentionally or inadvertently by human activity, and which has become established, becoming a permanent addition to the biota of the region. Other terms often used to refer to these species include "introduced", "exotic" or "alien" species that is established or has become "naturalized".

Non-native Invasive species (also known as 'alien invasive species' or simply 'invasive species'. The subset of 'established non-native species' (cf. definition above) that are recognized as causing negative consequences for the environment or for human activities and welfare.

If you answer "no" to this section's question, the questionnaire ends here.

2. A1: Have you published a regional species checklists in the last 20 years? *

Mark only one oval.

- Yes
- No

Section B - Personal background and self-assessment of expertise

Please provide some infomation about your scientific background

3. B1: For the following taxonomic groups indicate your self assessment of expertise *

Mark only one oval per row.

	No expertise	Little expertise	Moderate expertise	High expertise	Very high expertise
**Plants**	⬭	⬭	⬭	⬭	⬭
**Vertebrates**	⬭	⬭	⬭	⬭	⬭
**Invertebrates**	⬭	⬭	⬭	⬭	⬭
**Microorganisms**	⬭	⬭	⬭	⬭	⬭
**Fungi**	⬭	⬭	⬭	⬭	⬭

4. B2: For the following realms indicate your self-assessment of expertise *

Mark only one oval per row.

	No expertise	Little expertise	Moderate expertise	High expertise	Very high expertise
**Terrestial**	⬭	⬭	⬭	⬭	⬭
**Freshwater**	⬭	⬭	⬭	⬭	⬭
**Marine**	⬭	⬭	⬭	⬭	⬭

5. B3: For the following biomes indicate for which you published the checklists *

Mark only one oval per row.

	None	Very few	Some	Most	All
**Tropical**	⬭	⬭	⬭	⬭	⬭
**Subtropical**	⬭	⬭	⬭	⬭	⬭
**Temperate**	⬭	⬭	⬭	⬭	⬭
**Polar**	⬭	⬭	⬭	⬭	⬭

Section C - Species checklist research	Please provide some information about your scientific research that resulted in the publication of regional species checklists.

6. C1: How many species checklists have you published as author or co-author in the last 20 years? *

Mark only one oval.

- 1
- 2 to 3
- 4 to 5
- 6 to 10
- More than 10

7. C2: Please indicate your degree of focus on each of the different geographical scopes * indicated below.

Mark only one oval per row.

	No focus	Little focus	Moderate focus	Hight focus	Very high focus
**Species listing in human-dominated habitats (urban areas, farmlands, orchards, etc.).**	◯	◯	◯	◯	◯
**Species listing in protected areas (e.g., National Parks, Reservations, etc.).**	◯	◯	◯	◯	◯
**Species listing at the country level or for sub-national administrative divisions (e.g., Federal state, municipality, county).**	◯	◯	◯	◯	◯
**Species listing in non-protected and non-urban habitat (e.g., a wetland, forest, coastal habitat, etc.).**	◯	◯	◯	◯	◯
**Other typologies**	◯	◯	◯	◯	◯

8. C3: For the following taxonomic groups indicate for which you have published checklists *

Mark only one oval per row.

	None	Very few	Some	Most	All
**Plants**	◯	◯	◯	◯	◯
**Vertebrates**	◯	◯	◯	◯	◯
**Invertebrates**	◯	◯	◯	◯	◯
**Microorganisms**	◯	◯	◯	◯	◯
**Fungi**	◯	◯	◯	◯	◯

9. C4: According to the United Nations Geographical Subregions (Fig.S1.1), tell us of the region(s) of the globe in which you have published species checklists *

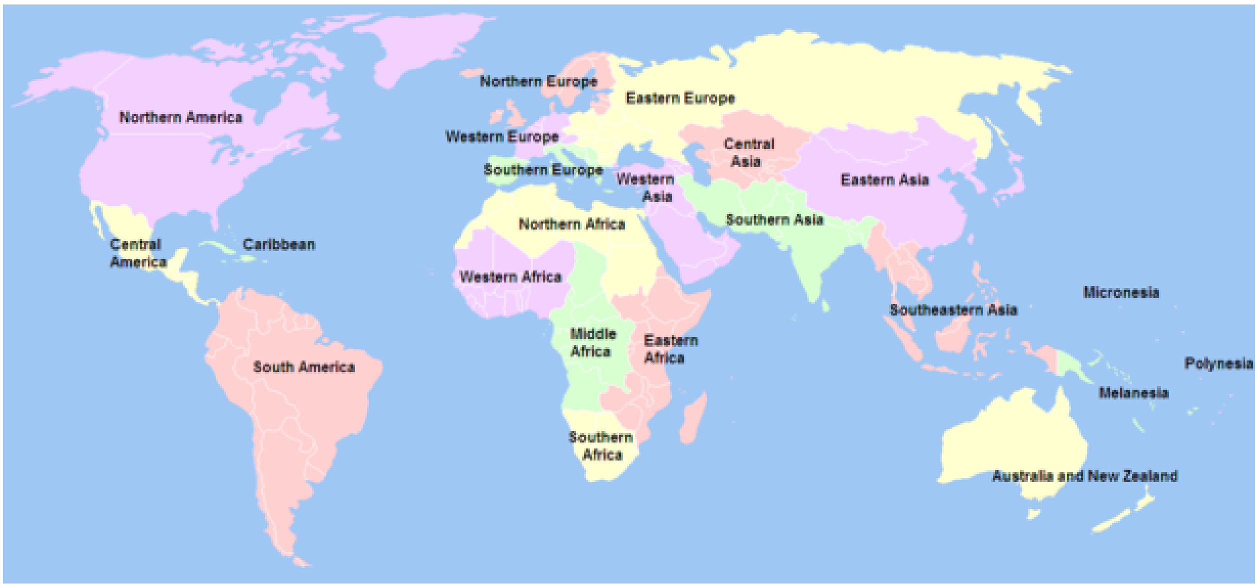

Fig. S1.1 - United Nations Geographical Subregions

Tick all that apply.

- [ ] Australia and New Zealand
- [ ] Caribbean
- [ ] Central America
- [ ] Central Asia
- [ ] Eastern Africa
- [ ] Eastern Asia
- [ ] Eastern Europe
- [ ] Melanesia
- [ ] Micronesia
- [ ] Middle Africa
- [ ] Northern Africa
- [ ] Northern America
- [ ] Northern Europe
- [ ] Polynesia
- [ ] South America
- [ ] Southeastern Asia
- [ ] Southern Europe
- [ ] Southern Africa
- [ ] Southern Asia
- [ ] Western Africa
- [ ] Western Asia
- [ ] Western Europe

Section D - Data content section

Please provide some information about the content in your checklists

10. D1: Concerning non-native established species, do you include these in the checklists you publish? *

Mark only one oval.

- Never
- Rarely
- Sometimes
- Very often
- Always

11. D2: If you answer "never" to "very often" in the question above, indicate the main reasons why you have not include them always. (If you answered "always" in the previous question, skip this question)

Mark only one oval per row.

	Never	Rarely	Sometimes	Very often	Always
**My work is not focused on non-native species**					
**No non-native established species was known for the listed area**					
**The amount of work/resources required to identify these species was too high**					
**Other reason**					

12. D3: If you include non-native species in your regional species checklists, do you list all you are aware of or just a subset of them? *

Mark only one oval.

- All
- The subset of those considered invasive (see definitions)
- A subset, but not obeying to a particular criterion
- Other
- Not applicable (I do not include these species).

13. D3.1: If you mentioned other reasons, please indicate them below.

____
____
____
____
____

14. D4: If you include non-native species in your regional species checklists, do you indicate their non-native status (i.e., do you differentiate these from native species)? *

Mark only one oval.

- Never
- Rarely
- Sometimes
- Very often
- Always

15. D5: Do you agree that omitting established non-native species from regional species lists or the indication of their non-native status is an obstacle to monitoring biogeographic and biodiversity change? *

Mark only one oval.

- Strongly agree
- Agree
- Neutral
- Disagree
- Strongly disagree

16. D6: Do you agree that the terminology on biological invasions and non-native species research, which comprises multiple terms and varying definitions, is an obstacle to the addition of non-native established species in regional species checklists? *

Mark only one oval.

- Strongly agree
- Agree
- Neutral
- Disagree
- Strongly disagree

17. D7: How knowledgeable are you about the terms and definitions used to refer to non-native species and invasive non-native species? *

Mark only one oval.

- No knowledge
- Little knowledge
- Moderate knowledge
- Good knowledge
- Expert-level knowledge

18. D8: Do you agree that the terminology on biological invasions and non-native species * is becoming increasingly standardised and well defined, so that the use of these terms in regional species control lists is increasingly straightforward?

Mark only one oval.

- Strongly agree
- Agree
- Neutral
- Disagree
- Strongly disagree

19. D9: If you include non-native species in the checklists and mention them as such, do * you also provide a definition of what the term means, or indicate a bibliographic source of reference for the definition?

Mark only one oval.

- Never
- Rarely
- Sometimes
- Very often
- Always
- Not applicable

Section E - Data delivery section

Please provide some information about how you provide the checklist data

20. E1: In your checklist publications, how do you supply the information to readers? *

Mark only one oval per row.

	Never	Rarely	Sometimes	Very often	Always
**Using descriptive text for each species**	⬭	⬭	⬭	⬭	⬭
**Using a standardised table or list summarising the same categories of information for all species**	⬭	⬭	⬭	⬭	⬭
**Other**	⬭	⬭	⬭	⬭	⬭

21. E2: If you include non-native species in your checklists, how do you indicate this status to readers? *

Mark only one oval per row.

	Never	Rarely	Sometimes	Very often	Always
**As descriptive text alongside the native species**	⬭	⬭	⬭	⬭	⬭
**As descriptive text in a specific section for non-native species**	⬭	⬭	⬭	⬭	⬭
**In a table alongside the native species (e.g., using symbols or other indications)**	⬭	⬭	⬭	⬭	⬭
**In a table or list specific for non-native species**	⬭	⬭	⬭	⬭	⬭
**I do not give this indication (not distinguishing native from non-native species)**	⬭	⬭	⬭	⬭	⬭

22. E3: In addition to the published checklists, do you also provide the data in a machine-readable format (e.g., excel file, CSV file, XML) or publish this data in a standardised biodiversity data repository? *

Mark only one oval.

- Never
- Rarely
- Sometimes
- Very often
- Always

23. E4: Do you agree that the structure of regional species lists, and the data formats used to publish them are becoming increasingly standardised? *

Mark only one oval.

- Strongly disagree
- Disagree
- Neutral
- Agree
- Strongly agree

This content is neither created nor endorsed by Google.

Google Forms
